# Academy of Stomatology

**Published:** 1901-07

**Authors:** 

**Affiliations:** Academy of Stomatology


### Reports of Society Meetings.

### ACADEMY OF STOMATOLOGY.

A REGULAR meeting of the Academy of Stomatology of Philadelphia was held at the rooms of the Academy, 1731 Chestnut Street, on the evening of Tuesday, April 22, 1901.

In the absence of the president and vice-president, Dr. E. T. Darby occupied the chair as president *pro tem.*

*The President.*—As Chairman of Council, I would report that the essayist of the evening has failed to present himself, for some reason unknown to the Council.

In the absence of the essayist and his paper to-night, a short paper will be read by Dr. J. C. Curry.

*Dr. Curry.*—Mr. President and Members of the Academy, I think it is hardly fair to dignify my contribution by calling it a paper. As I had no idea of saying anything until this morning, and did not find out clearly just what I was going to say until to-night, and am not quite sure of the matter now, I will try to reward your indulgence by being brief. The subject that I wish to bring before you is that of quack dentistry, and I would say at the start that I shall take it for granted that every legitimate dentist is entirely competent to take care of himself, and I will speak for the public only.

Within the past ten years there has been an influx of quack dentists, who are becoming more numerous, daring, and dangerous, and it seems to me impossible for our educational institutions to compete with these pests unless they have the hearty co-operation of every organized body of dentists, as well as of every individual dentist who has the welfare of the public and his profession at heart. Neither the colleges nor the State boards can make a man honest, and the man who graduates from a college a skilful rogue soon becomes a conscienceless charlatan. He is absolutely unscrupulous in obtaining the confidence of the people and their money; he is careless in his methods, and resorts to malpractice in most cases. Of course, the natural reply to this would be that the public should know better, but how can people know better unless they have been taught, and who is better able to teach them than we who know? They themselves find it out after it is too late and after they have suffered keenly. We must remember that the people who patronize these places are for the most part, though not entirely, from the middle and lower classes, who are driven by actual necessity and are hampered by lack of time and money. They are, for the most part, ignorant of our profession, because they have never been taught. How can they be otherwise? I believe it to be the duty of the dental profession to devise means of reaching the public and stopping the wholesale malpractice which every one of us here knows is going on uninterruptedly, every day, Sundays and evenings included. It is not enough to say that we have dental colleges for the protection of the public against unskilled professional men, or that we have State boards to support the colleges. It is not lack of skill I am complaining of, but it is the lack of principle with which these institutions, the advertising offices, are carried on. The average individual who goes about his work from seven o'clock in the morning to six at night has not time to investigate their methods, nor the time to go to a dentist and make an appointment, and in the majority of cases he cannot afford the expense of first-class work. He is shown the advertisements of these quacks and led in a number of ways to believe that they are public benefactors, and he goes to them. In the majority of cases, however, he is very sorry for it all his life.

We have all sorts of safeguards thrown about the public, but I believe that at the present time there is no way to reach the unscrupulous quack dentist. The people who go to these establishments to get services are fleeced of their money, and when they make complaint they are laughed at and told to go to some place else, or anything that happens to come to the mind of the proprietor. They cannot afford to bring a civil suit for damages, and the result is they pocket their losses, and their teeth usually, and go their ways sorrowing.

I would simply bring this subject before our Society for discussion. There usually is a way to right most of the wrongs. There surely is a way of reaching an intelligent public. We believe the American people is an intelligent people, and I think there ought to be a concerted movement all over the country to stop this deception. If I should speak from the stand-point of the dentist, I would appeal to you through your professional pride. I would say to you that it does not make a dentist feel very proud to be introduced as a dentist, when Dr. So-and-so is advertising to sell teeth at one dollar and ninety-eight cents per set. I would also say that the young men who are graduating from our schools to-day are thrown out upon the large cities and have to compete with these men. They have neither prestige nor experience to fall back on, and a great many of them have not very much money, and thus many of them are driven into the offices of these quacks as assistants. There is not a living for them unless they do go into advertising offices.

### DISCUSSION.

*Dr. Kirk.*—I think that the subject is a very important one, but whenever this topic comes up, either for discussion in any dental meeting that I have attended or whether it comes up in the journals, I am always reminded of the attitude of Abraham Lincoln towards the man who was persistently requesting an audience with the President, and finally succeeded in getting in, and then read to him an original poem some several pages long, dealing with the beginning and end of the universe, and so on, which he read with a great deal of impressiveness; and when he finished he said,—

"Now, Mr. President, will you tell me what you think about it?"

And it is said that Mr. Lincoln considered the matter very carefully and replied,—

"Well, if anybody likes that kind of thing, that is just about the kind of thing they like."

Now, the application of this matter to the present case is this: The people that do not know any better than to patronize advertising establishments make very undesirable patients. I had more or less a feeling of gratitude myself that there were such places, as it furnished an outlet for the patients that I personally did not want to have.

I have no doubt that a great deal of harm is done and that a great many teeth are slaughtered, and no doubt that, in the aggregate, a great amount of money is taken away from an unsuspecting public. On the other hand, we have the evidence, in a few cases, that some of these men who are what might be called "quacks," while they are doing an advertising business and not advancing the profession, are endeavoring to conduct their practices upon business principles. There was a man who died not long ago who was noted for the fact that he did carry on an honest advertising business and had amassed a considerable competence. I mention that on account of what I believe to be true,—*i.e.*, that the advertiser is not necessarily an irresponsible quack. Just how the evil is to be cured I do not know. I do not know of a better suggestion than the one made by the essayist, that of educating the public. As to the details of the plans of doing that successfully, I must confess my inability just at the present time to see any method by which that could be properly done. Dr. Curry suggests an organized movement, an organized attack upon this difficulty throughout the country. I should be glad to know just what he means by that. There is always the danger in such an organized attack of putting these men on the defensive and in the position of being persecuted, and thus arousing public sympathy on their side. It is a very delicate matter to handle, and the desired end may be defeated by awakening that kind of sentiment against the individuals who are the attacking party.

Unfortunately the press of the country is almost solidly in favor of these men, simply because they derive large incomes from their advertisements. It is very difficult to get any paper, I think, to take up a warfare against these men. They want some better reason than we are able to give that it is unprofessional before they will cut off a fairly good source of income. The problem seems to be insoluble in so far as the details of doing the work is concerned, and yet I believe that the difficulty is to be met only as has been suggested by the essayist, by proper education of the public, so that they may be brought up to a proper appreciation of what good dental service means.

*Dr. Ellerbeck.*—I have not much to say on this subject, but I view it as presented by Dr. Curry particularly from the side that relates to the public and very little from the aspect of the quack dental parlor. We know that that subject has been discussed through the journals since the beginning of the formation of dental societies, without a great deal of good. As Dr. Curry and Dr. Kirk have said, the matter rests with the education of the public. As to the proper method of bringing about that education, it seems to me it can only be done through the education of the school children, who should be taught the proper care of their teeth and, incidentally, the right place to go to get proper treatment.

I would like to hear a discussion of the possibility of having examining dentists in the public schools, and of the best means of bringing that about throughout the country generally.

*Dr. Brubaker.*—I would like to ask one question. What reasons can be assigned for the rapid increase of commercial dentistry in this country? I would like to hear from some of the older members of the profession.

*Dr. James Truman.*—Before the inauguration of State boards in this country we had quacks, but they were limited in number and in power. At the time that the State boards were organized throughout the country these very same men evidently made up their minds that another method must be adopted in order to overcome the statute. They organized these so-called "dental parlors," and, instead of running them themselves, with perhaps some inefficient assistants, they called to their aid young men who had just graduated, who, being without much means, perhaps none at all, in order to get along, were willing to accept the positions under these men. Having those young men with them, they did a better class of work. I am not prepared to say that the work that is done in these parlor establishments is not quite as good as that done in the clinics of our colleges.

I know nothing about them further than that they began practically with the establishment of laws regulating dentistry, and have increased just in proportion to the severity of those laws, and not only increased in this State, but have increased in every State of the Union, and in almost every town. I do not know that the application of law would help the matter at all. I see no help for it. I question very much whether the education of the children of the public schools, while it would be a great advantage unquestionably, would teach these children to go to the proper dentist. The general feeling is always to get things as cheap as possible. This is seen in the stores, where the bargain-counter is crowded all the time, even when people know they are getting an inferior article. So it will always be with these parlor establishments, or establishments where they advertise a cheap article. I see nothing for it but a proper education of the people as to what is good dentistry and what is not, and I question very much whether that would accomplish a great deal.

*Dr. Kirk.*—If I understand Dr. Truman correctly, he reasons that this increase in the advertising dental parlors, which came after the creation of the State boards, is because of the establishment of the State boards. I cannot see any good reason why the establishment of legal restrictions for the practice of dentistry should create an interest in the business of these dental parlors. There was also another increase,—namely, a tendency of men throughout the country to take up the study of dentistry, and a large multiplication of dental schools. I can see more reason why they were the result of State board creation than the dental parlor, but I do not see any connection between the laws limiting the practice of dentistry and the development of these men who are carrying it on apparently illegally.

*Dr. James Truman.*—I can see a direct connection. The State law has driven these men to get young men from colleges into their offices, with the result that they have created a certain degree of confidence among the people generally in their establishments which did not exist before. That is directly traceable to the force of law.

*Dr. Roberts.*—The reason for the increase in advertising dental parlors is the desire to make money and the large amount of unemployed capital seeking investment. The financiers who have money see a scheme for making money; therefore they start in with these dental parlors, and they will do it with any business or any profession they can. It seems unfair to attribute the increase in advertising parlors to the formation of dental examining boards. All the dental examining boards can do, and all they have done, is to say whether those desiring to practise in a State have received a certain amount of education or have the requisite fitness.

*Dr. Brubaker.*—My reasons for asking the question was simply to get opinions from many members of the society, because it is very evident that commercial dentistry is increasing with enormous rapidity, not only here, but in every city in the State, and the question, therefore, arises, in connection with any kind of control of these people, What are the causes that have led to it? It is impossible to try to treat a disease without knowing the cause. There must be a cause for this condition. I do not know what it is or what the causes may be. Personally I have thought for a long time that it is part and parcel of the general commercial spirit of the age in which we are living. There seems to be a tendency towards the formation of trusts on a small scale, to try to regulate the prices of dental work, and so on. But the "dear public" does not wish to be saved any more physically than it does morally, and, therefore, I do not place much faith in the education of the people. Medical men have tried to educate the people for a long time, but they have not been successful, and the dental people will find very much the same thing. Other causes are at work, and unless they are eradicated it will increase. Naturally, therefore, I was somewhat curious to know what causes have combined to produce the present condition. I know this, and probably everybody else does, that there is a bait held out every year for a certain number of graduates of dental colleges to enter these establishments. They are being organized rapidly and offer fairly good wages, and they solicit men to go into their establishments. Young men having the commercial spirit well developed or inherited take up tne work. That does not account for the existence of the parlors.

*Dr. Curry.*—Having been asked what I mean by "organized attack," I would say, that is a question which I have come here to ask rather than to answer, and inasmuch as I have declared at the start that my contribution was not a paper, I did not bring any remedy, but would say this: We are taught in the colleges that the aim of the college is to educate the man, and it is necessary that he be educated if the public is to be protected, for he will serve the public, and unless he is well educated he serves them ill. That being the case, why cannot the law which authorizes colleges and compels students to take the course of study prescribed by them before they are granted a diploma go one step farther and make the dental college responsible for the professional conduct of graduates after they have left the institution? If a lawyer goes into any

"shyster" business, and shows himself unprincipled or unskilled, his commission is taken from him and he cannot practise law. Now, you could get at this by compelling every operator in every bogus college, school, or institution to have his diploma before his chair, in plain sight; then if there is any question about the man's ability or his principles, the college which graduated that man can be informed of it and his diploma can be revoked, if necessary legislation were enacted. In reference to the argument against prosecutions, that same principle could be applied to the State examining boards. I know the men who graduate are put to a great deal of trouble and expense to take the State board examinations. If they do not take them they are prosecuted. You might just as well say we could not prosecute them, because they would have the sympathy of the community when such prosecution was brought.

An organized attack could be brought in this way: Each dental society, State board, and college could appoint its corresponding secretary as committeeman to correspond with others and get a consensus of opinion on this question, and then they in turn could request the local boards of education to appoint a lecturer in their schools to lecture to the more advanced classes, and teach them not only in regard to dentistry, but also warn them against taking quack medicines. I think it is too bad that we are so very strenuous on some points,—for instance, in the matter of food products, —when, on the other hand, for ten cents any one can without trouble buy enough laudanum or arsenic to send a family to perdition.

Dr. Brubaker asks the reason for the increase in advertising dentists. The question has been answered,—"It pays." They advertise to make a set of teeth for one dollar and ninety-eight cents. You go to get a set of them, and they bring you a set and say, "We have these for colored people, and we would not want you to take them, but for fifteen dollars we can make you something that is worth while," and the "dear public" is perfectly willing to be gulled, and goes on to buy something "worth while." Now, it may be that they do not want to be saved from the hands of these sharks. Yet the duty is ours, none the less; if we are to represent the dental profession with any degree of pride and truth and give it our best work and our best thought, we must bring it up to a certain standard. We cannot shirk the responsibility by saying, "People don't want to be protected."

All dentists who have the best interests of their profession at heart should combine and say to patients when they apply for cheap service, " My time is worth more than you can pay for; but in this city we have dental colleges, and you can go to the college which is the nearest to you and even if you have a student to work for you who is not a graduate, you have a man who is under the direction of skilful operators, and you will be honorably dealt with." This question does not affect me personally, as I have enough work to do, all that I can comfortably do, but with the interest of my profession at heart, I want to do all I can to elevate it, and do not wish to be associated in name even with those who are doing all they can to destroy the confidence of the people.

I am afraid I have spoken too long, and am very sorry the discussion of this subject was not taken up more fully.

Adjourned.

OTTO E. INGLIS, D.D.S.,
*Editor Academy of Stomatology.*

